# Comparative Resistance of AH26 and a New Sealer Prototype to a Bacterial Challenge

**DOI:** 10.1155/2012/365231

**Published:** 2012-02-22

**Authors:** Derek Duggan, Sheng Zhong, Eric Rivera, Roland Arnold, Eric Simmons

**Affiliations:** ^1^Department of Endodontics, UNC School of Dentistry, Chapel Hill, NC 27599, USA; ^2^Diagnostic Sciences and General Dentistry, UNC School of Dentistry, Chapel Hill, NC 27599, USA; ^3^Dental Research Center, UNC School of Dentistry, Chapel Hill, NC 27599, USA

## Abstract

*Objective*. This study compared the leakage resistance of a New Sealer Prototype (NSP) with a traditional sealer (AH 26) in Resilon-filled roots subjected to a bacterial challenge. *Study Design*. 41 roots were instrumented to ISO size 50 apically. Group 1 (*n* = 20) contained Resilon and AH 26 sealer and roots in group 2 (*n* = 21) contained Resilon and NSP. Roots were embedded in a dual-chamber model with the upper chamber containing *Streptococcus mutans* inoculum. Evidence of bacterial penetration was observed for 1 month. Fisher's Test was used to analyze the data. *Results*. 8 of 20 roots (40%) in the AH 26 group demonstrated leakage whereas 3 of 21 roots (14%) in the NSP group leaked. The difference in leakage rates was not statistically significant (*P* = 0.053). *Conclusion*. The traditional sealer (AH 26) demonstrated increased leakage rates compared to the New Sealer Prototype (NSP), but the difference did not reach statistical significance in this study.

## 1. Introduction

The presence of bacteria in a root canal system is a pre-requisite for the development of apical periodontitis [1–4]. Following biomechanical instrumentation, a root canal filling aims to seal the root canal system as optimally as possible, preventing bacteria and/or nutrients from moving into or out of the root canal system. If this is achieved, then healing of periapical tissues should occur if an adequate coronal seal is present [5]. 

 Although the bulk of the root canal system is commonly filled using a solid core material, a sealer is used to occupy remaining gaps in the root canal system. Even in the presence of a sealer, gutta-percha, the traditional core material used in endodontics, will invariably allow bacteria to penetrate the entire length of root canal systems [6–9]. A wide range of sealers are used in endodontics including zinc-oxide-eugenol-based, silicone-based, calcium hydroxide-based, and resin-based varieties. Leakage resistances of different sealers have been tested with a variety of *in vitro* leakage models, with individual sealers exhibiting variable resistance to the penetration of a range of test materials [10–15]. However, the validity of such leakage experiments has been called into question [16, 17]. 

Nevertheless, if new sealers are to be used in humans, it is important to subject such materials to appropriate tests to ensure that they meet minimum requirements. *In vitro *leakage tests are a useful screening tool to test such materials. The bacterial leakage model consists of an upper chamber containing one or more microbial species and a lower chamber containing a sterile medium, a fermentable sugar, and a pH indicator. A root is sealed into position between the upper and lower chambers in such a way that the only way in which bacteria can enter the lower chamber is through the root canal itself. When bacteria pass into the lower chamber, a pH indicator in the lower chamber will change color as the fermentable sugar is metabolized by the pathogen.

In recent years, resin-based core materials and complementary sealers have been introduced, bringing composite bonding technology into the field of endodontics. The stated objective of these obturating materials is to enhance the leakage resistance of root canal fillings by creating a chemomechanical bond between the core filling and the root canal walls. Resilon is a synthetic polymer-based root filling material used in combination with a resin-based composite sealer. Resin-based sealers are being continuously developed and tested and claim to offer improved bonding capabilities within the root canal system. Several *in vitro *and animal leakage studies have demonstrated the ability of resin-based sealers to better resist bacterial penetration compared to conventional sealers [18–21]. A New Sealer Prototype (NSP), a self-etching resin sealer, has been developed by Sybron Dental Specialties to be used in conjunction with Resilon. The purpose of this study was to compare the ability of NSP and AH 26 sealers to resist bacterial penetration in Resilon-filled roots using a two-chamber bacterial leakage model. The null hypothesis is that there is no statistically significant difference between the two tested sealers regarding their ability to resist bacterial penetration through Resilon-filled roots.

## 2. Materials and Methods

A total of 41 single-rooted adult human incisor and canine teeth extracted in a University Oral Surgery Department were used in this study. The teeth were stored in 0.2% thymol in normal saline solution until required. Following storage, all teeth were placed in 5.25% sodium hypochlorite (NaOCl) for 15 minutes to remove organic soft tissue from the root surfaces. Any tissue remaining after 15 minutes was gently removed with a curette. 

The crowns were removed at the CEJ level and the roots were shortened to approximately 16 mm in length from the coronal end using a high-speed handpiece and Endo-Z burs (Dentsply Maillefer, Tulsa, OK, USA). A dental operating microscope (Global Surgical Corp., St. Louis, MO, USA) was used to visually inspect the root surfaces for cracks under 8 times magnification.

Stainless steel K-files (Size 06–15) were manipulated apically until the tip of each file was visible at the apical extent of each canal. The working length was established 1 mm short of this length. Rotary instrumentation was performed with a crown-down technique using K3 nickel titanium rotary files (SybronEndo Corp, Orange, CA, USA). The last rotary file taken to length in all roots was an ISO size 50 file of 0.04 taper. A total of 15 mL of 1.25% sodium hypochlorite was used during instrumentation to irrigate each root canal using a 10 mL syringe and a 30-gauge nickel titanium needle (Vista Dental Products, Racine, WI, USA). Following the instrumentation phase, 2 mL of 17% Ethylenediaminetetraacetic acid (Vista Dental Products, Racine, WI, USA) was used for 1 minute to remove the smear layer. This was followed by a final flush of 2 mL of 2% Chlorhexidine solution for 1 minute (Vista Dental Products, Racine, WI, USA). Each canal was dried with paper points (SybronEndo Corp, Orange, CA, USA) and filled using one of two protocols.

**Group 1 [Lateral Compaction of Resilon with AH26 Sealer]**

A master cone (either an ISO size 50 cone or an ISO size 45 cone shortened to fit apically if required) was coated with AH 26 sealer (Dentsply Maillefer, Tulsa, OK, USA) and placed to working length in the canal. A fine finger spreader (Dentsply Maillefer, Tulsa, OK, USA) was then inserted into the canal to permit placement of fine accessory cones. Successive accessory cones were coated with AH 26 and placed in each canal until the finger spreader did not penetrate past the coronal third of the root with moderate digital pressure.

**Group 2 [Lateral Compaction of Resilon with NSP]**

Group 2 roots were filled using an identical protocol to that used in Group 1, except that NSP was used in place of AH 26 sealer.

Teeth were placed in an incubator for one week at 37 degrees Celsius to allow the sealers to set. A two-chamber leakage model as described by Shipper et al. [22] was used. The upper chamber consisted of a Corning 15 mL polycarbonate centrifuge tube (Corning Inc., Corning, NY, USA) with a small hole prepared at the bottom. Each root was gently pushed through the opening from the tube's interior until approximately one half of it protruded through the end of the tube. Lateral residual space between the tube and each root was sealed with soft rope utility wax. Additional heated wax was painted over the apical root surface except for the apical foramen. This was done to ensure that the only potential passage through the root into the lower chamber was through the root canal. *Streptococcus mutans *strain NCTC 10449 (serotype C) was used to test the susceptibility of the root filled canals to bacterial leakage.

Each upper chamber and its screw cap were sterilized separately using Ethylene Oxide gas and subsequently reattached. The lower portion of the upper chamber was then inserted into a scintillation vial, which constituted the lower chamber of the leakage set-up. The junction between the upper chamber and the scintillation vial was sealed using additional heated soft rope utility wax. Prior to the start of the experimental phase, test dual-chamber models with embedded teeth were constructed and tested for sterility, confirming the integrity of the experimental model.

A volume of 9 mL of media inoculated with exponentially growing *S. mutans 10449 *was added to the upper chamber at the start of the 1-month observation period. This was considered day zero (September 18). The upper chambers were monitored daily by one of the authors (E. Simmons) for visible (cloudy) growth. The upper chambers were refreshed with exponentially growing batch cultures every three days until day 17 (October 5), and the lower chambers were monitored for color change (red to orange to yellow) and bacterial growth (clear to cloudy) daily. Cloudy lower chambers were plated to mitis-salivarius-bacitracin (MSB) agar to confirm recoverable colonies with morphology consistent with *S. mutans*. Chamber content was also plated to sheep blood agar to assure a lack of contamination.

After day 20 (October 8), the upper chamber was replenished with fresh uninoculated media. All but one upper chamber established growth without further inoculation and media was refreshed through day 27 (October 15). The upper and lower chambers of all set-ups were cultured to MSB agar on day 30 (October 18) and determined to be positive or negative for recoverable *S. mutans. *

The medium was not replenished in the lower chamber at any stage during the study. The lower chamber medium is a minimal medium that has sucrose as the only fermentable sugar to help confirm *S. mutans *metabolism. It is rapidly depleted once bacteria start to grow and thus would not be expected to maintain the viability of *S. mutans *for very long. This was indeed the case as some cultures taken on initial evidence of bacterial growth were positive for *S. mutans *but by day 30 were negative for recoverable *S. mutans*. Leakage was therefore considered positive if there were recoverable *S. mutans *from the lower chamber at either time point.

## 3. Results

Three specimens in Group 1 (AH26) and two specimens in Group 2 (NSP) were contaminated during the preparatory phase of the leakage experiment and were discarded. This left a total of 20 units in the AH 26 group and 21 units in the NSP group.

Fisher's Exact Test was used to compare leakage rates in the 2 groups. Differences were considered significant if the *P* value was less than 0.05. After 1 month, 8 out of 20 root canals sealed with AH 26 allowed leakage of *S. mutans*. Evidence of leakage through individual roots in the AH 26 Group is presented in Table 1. In contrast, 3 out of 21 of the root canals sealed with NSP demonstrated evidence of leakage. Evidence of leakage through individual roots in the NSP Group is presented in Table 2. Although the NSP group leaked less than the AH 26 group, the difference was not statistically significant (*P* = 0.053).

## 4. Discussion

*In vitro* leakage studies cannot replicate the complex *in vivo* environment, where leakage of microbes involves a dynamic interplay between the obturated canal system, a highly evolved microbial community, and a fully functional immune defense system in the periradicular tissues. They do, however, indicate the relative resistance of one sealing material compared to another when exposed to a particular pathogen/subset of pathogens. *S. mutans*, the test pathogen used in this study, is a Gram positive aerotolerant anaerobe which commonly inhabits the oral cavity. Furthermore, a high proportion of bacterial species isolated from obturated root canals in failed cases are aerotolerant by nature. Therefore, using such a test pathogen in this study would seem to be clinically relevant. 

A change in the color of the sucrose-containing broth in the lower chamber from red to yellow (due to the presence of pH indicator) would indicate the presence of sucrose-fermenting *S. mutans* as lactic acid is produced (Tables 1 and 2). This would demonstrate that the streptococcus species in the upper chamber passed through the root-filled teeth and into the lower chambers. Although several roots demonstrated negative cultures in the lower chamber at the endpoint of the experiment, roots demonstrating a color change had to exhibit a positive culture at the time of the color change to be considered to be definitively leaking. Although every effort was made to minimize the risk of contamination of the lower chambers, the possibility of external contamination during the experimental period cannot be discounted. Another possible avenue for bacteria to enter the lower chamber would have been through a visually undetected crack in the roots and/or between the wax seal and the corning tubes. 

NSP is an adhesive monomer compatible for use with Resilon. It contains an acidic methacrylate resin, which is claimed by the manufacturer to permit bonding without separate etching and bonding procedures. The lower leakage rates when using a resin-based sealer in this study were observed in an earlier study comparing resin-based and traditional sealers [22]. However, the difference in leakage rates between sealer types was not statistically significant in this study. Questions remain regarding the integrity of the bond between self-etching resin-based sealers and the root wall [23–25]. The optimal irrigation protocol to ensure maximal polymerization when using a resin-based sealer is another area that requires further research [26]. 

The fact that there was a trend towards statistical significance when comparing the leakage rates between the groups suggests that sealers such as NSP merit further investigation. The potential antibacterial effect of several sealers has previously been studied [27, 28]. In light of the challenges in establishing a viable upper chamber inoculum in the presence of NSP in this study, it is proposed that further research be conducted to investigate the antibacterial properties of NSP. It may be that the observed antibacterial effect NSP was instrumental in this group of teeth demonstrating comparatively low leakage rates. However, at present, this hypothesis is only conjectural.

## 5. Conclusion

Although Resilon-filled root canals sealed with AH 26 were less effective than those sealed using NSP in preventing leakage of *S. mutans, *this difference was not statistically significant (*P* < 0.05). Therefore, the null hypothesis that leakage rates are similar between the two sealer groups is accepted. 

## Figures and Tables

**Table 1:** Color change and culture findings from lower chamber in AH26 Group.

Unit*	Color^¶^	Day^†^	Positive culture^‡^	Leakage^§^
1	Red	None	No	No
2	Yellow/orange	30	Yes	Yes^§^
3	Yellow	9	Yes	Yes^§^
4	Red	None	No	No
5	Yellow/orange	19	Yes	Yes^§^
6	Orange	15	Yes	Yes^§^
7	Red	None	No	No
8	Orange	25	No	No
9	Red	None	No	No
10	Yellow	4	Yes	Yes^§^
11	Red	None	No	No
12	Red	None	No	No
13	Yellow	4	Yes	Yes^§^
14	Red	None	No	No
15	Yellow	13	No	Yes^§^
16	Orange	26	No	No
17	Orange	15	No	No
18	Red	None	No	No
19	Orange	30	No	No
20	Yellow	18	No	Yes^§^

*Each unit consists of a dual-chamber model with an embedded root between the chambers.

^¶^Color in the lower chamber after 30 days (Red: no change; yellow: acid; orange: weakly acidic).

^†^Day when a color change was first observed in the lower chamber.

^‡^Cultivable *S. mutans* from lower chamber sample after 30 days.

^§^Evidence for bacterial penetration through root canal into lower chamber.

**Table 2:** Color change and culture findings in lower chamber of NSP Group.

Unit*	Color^¶^	Day^†^	Positive culture^‡^	Leakage^§^
1	Yellow	4	Yes	Yes^§^
2	Red	5	No	No
3	Red	20	No	No
4	Orange	18	No	No
5	Orange	27	No	No
6	Orange	24	No	No
7	Yellow	5	Yes	Yes^§^
8	Yellow	19	Yes	Yes^§^
9	Orange	16	No	No
10	Orange	27	No	No
11	Red	17	No	No
12	Red	None	No	No
13	Red	17	No	No
14	Orange	19	No	No
15	Red	None	No	No
16	Red	None	No	No
17	Orange	16	No	No
18	Orange	18	No	No
19	Red	None	No	No
20	Red	None	No	No
21	Red	None	No	No

*Each unit consists of a dual-chamber model with an embedded root between the chambers.

^¶^Color in the lower chamber after 30 days (Red: no change; yellow: acid; orange: weakly acidic).

^†^Day when a color change was first observed in the lower chamber.

^‡^Cultivable *S. mutans* from lower chamber sample after 30 days.

^§^Evidence for bacterial penetration through root canal into lower chamber.

## References

[1] Kakehashi S, Stanley HR, Fitzgerald RJ (1965). The effects of surgical exposures of dental pulps in germ-free and conventional laboratory rats. *Oral Surgery, Oral Medicine, Oral Pathology*.

[2] Bergenholtz G (1974). Micro organisms from necrotic pulp of traumatized teeth. *Odontologisk Revy*.

[3] Sundqvist G (1976). *Bacteriological studies of necrotic dental pulps*.

[4] Moller AJR, Fabricius L, Dahlen G (1981). Influence on periapical tissues of indigenous oral bacteria and necrotic pulp tissue in monkeys. *Scandinavian Journal of Dental Research*.

[5] Ray HA, Trope M (1995). Periapical status of endodontically treated teeth in relation to the technical quality of the root filling and the coronal restoration. *International Endodontic Journal*.

[6] Swanson K, Madison S (1987). An evaluation of coronal microleakage in endodontically treated teeth. Part I. Time periods. *Journal of Endodontics*.

[7] Torabinejad M, Ung B, Kettering JD (1990). In vitro bacterial penetration of coronally unsealed endodontically treated teeth. *Journal of Endodontics*.

[8] Khayat A, Lee SJ, Torabinejad M (1993). Human saliva penetration of coronally unsealed obturated root canals. *Journal of Endodontics*.

[9] Friedman S, Komorowski R, Maillet W, Klimaite R, Nguyen HQ, Torneck CD (2000). *In vivo* resistance of coronally induced bacterial ingress by an experimental glass ionomer cement root canal sealer. *Journal of Endodontics*.

[10] Almeida JFA, Gomes BPFA, Ferraz CCR, Souza-Filho FJ, Zaia AA (2007). Filling of artificial lateral canals and microleakage and flow of five endodontic sealers. *International Endodontic Journal*.

[11] Belli S, Ozcan E, Derinbay O, Eldeniz AU (2008). A comparative evaluation of sealing ability of a new, self-etching, dual-curable sealer: hybrid Root SEAL (MetaSEAL). *Oral Surgery, Oral Medicine, Oral Pathology, Oral Radiology and Endodontology*.

[12] Bouillaguet S, Shaw L, Barthelemy J, Krejci I, Wataha JC (2008). Long-term sealing ability of Pulp Canal Sealer, AH-Plus, GuttaFlow and Epiphany. *International Endodontic Journal*.

[13] Yilmaz Z, Tuncel B, Ozdemir HO, Serper A (2009). Microleakage evaluation of roots filled with different obturation techniques and sealers. *Oral Surgery, Oral Medicine, Oral Pathology, Oral Radiology and Endodontology*.

[14] Camilleri J, Gandolfi MG, Siboni F, Prati C (2011). Dynamic sealing ability of MTA root canal sealer. *International Endodontic Journal*.

[15] Oliveira ACM, Tanomaru JMG, Faria-Junior N, Tanomaru-Filho M (2011). Bacterial leakage in root canals filled with conventional and MTA-based sealers. *International Endodontic Journal*.

[16] Schuurs AH, Wu MK, Wesselink PR, Duivenvoorden HJ (1993). Endodontic leakage studies reconsidered—part II. Statistical aspects. *International Endodontic Journal*.

[17] Wu MK, Wesselink PR (1993). Endodontic leakage studies reconsidered—part I. Methodology, application and relevance. *International endodontic journal*.

[18] Shipper G, Teixeira FB, Arnold RR, Trope M (2005). Periapical inflammation after coronal microbial inoculation of dog roots filled with gutta-percha or resilon. *Journal of Endodontics*.

[19] Leonardo MR, Barnett F, Debelian GJ, de Pontes Lima RK, Bezerra da Silva LA (2007). Root canal adhesive filling in dogs’ teeth with or without coronal restoration: a histopathological evaluation. *Journal of Endodontics*.

[20] Pereira CC, de Oliveira EP, Gomes MS (2007). Comparative *in vivo* analysis of the sealing ability of three endodontic sealers in dog teeth after post-space preparation. *Australian Endodontic Journal*.

[21] Eldeniz AU, Ørstavik D (2009). A laboratory assessment of coronal bacterial leakage in root canals filled with new and conventional sealers. *International Endodontic Journal*.

[22] Shipper G, Ørstavik D, Teixeira FB, Trope M (2004). An evaluation of microbial leakage in roots filled with a thermoplastic synthetic polymer-based root canal filling material (Resilon). *Journal of Endodontics*.

[23] Schwartz RS (2006). Adhesive dentistry and endodontics—part 2: bonding in the root canal system—the promise and the problems: a review. *Journal of Endodontics*.

[24] Babb BR, Loushine RJ, Bryan TE (2009). Bonding of self-adhesive (self-etching) root canal sealers to radicular dentin. *Journal of Endodontics*.

[25] Kim YK, Mai S, Haycock JR (2009). The self-etching potential of RealSeal versus RealSeal SE. *Journal of Endodontics*.

[26] Wu WC, Shrestha D, Wei X, Ling JQ, Zhang WH, Chen J (2010). Degree of conversion of a methacrylate-based endodontic sealer: a micro-Raman spectroscopic study. *Journal of Endodontics*.

[27] Slutzky-Goldberg I, Slutzky H, Solomonov M, Moshonov J, Weiss EI, Matalon S (2008). Antibacterial properties of four endodontic sealers. *Journal of Endodontics*.

[28] Kayaoglu G, Erten H, Alaçam T, Ørstavik D (2005). Short-term antibacterial activity of root canal sealers towards Enterococcus faecalis. *International Endodontic Journal*.

